# An Account of the Cases of Dislocation of the Femur at the Hip-Joint Treated by Manipulation Alone, after the Plan Proposed by W. W. Reid, M. D., of Rochester, Which Have Occurred in the New York Hospital during the past Two Years

**Published:** 1855-03

**Authors:** 


### ECLECTIC DEPARTMENT,

### AND SPIRIT OF THE MEDICAL PERIODICAL PRESS.

---

*An account of the Cases of Dislocation of the Femur at the Hip-joint treated by Manipulation alone, after the plan proposed by* W. W. Reid, M. D., *of Rochester, which have occurred in the New York Hospital during the past two years.* By Thomas M. Markoe, M. D., one of the Attending Surgeons.

In the "Transactions of the Medical Society of the State of New York, during its annual session held at Albany, February 3d, 1852," is published a paper by William W. Reid, M. D., of Rochester, N. Y., on "Dislocation of the femur on the dorsum ilii, reducible without pullies or any other mechanical power," in which it is the object of the writer to show that this displacement can be remedied "by flexing the leg on the thigh, carrying the thigh over the sound one, upward over the pelvis, as high as the umbilicus, and then by abducting and rotating it."

The fact that luxations of the hip-joint might be reduced by manipulations, not requiring forcible extension by pullies or other mechanical contrivance, seems to have presented itself to the mind of several writers both of ancient and of modern times. The idea would appear sometimes to have been suggested by accident, and sometimes to have been the result of reasoning on the mechanism of the joint and its displacements. Such a suggestive accidental reduction occurred in the hands of Dr. Physick, and is related in Dorsey's Surgery. A patient was the subject "of dislocation of the femur directly backward, and after very powerful efforts had been made to effect the reduction by extension, Prof. Physick, conjecturing that the head might be confined in a slit in the capsular ligament, discontinued the extending force entirely, and then, with no more force than that of his own hands, abducted the thigh, when the bone instantly slipped into its place."

A similar occurrence took place in our hospital, about the time of the introduction of the use of chloroform, in a patient with dislocated hip, under the care of Dr. John C. Cheesman. While the preparations for applying mechanical force were being made, Dr. C. made some preliminary movements of the joint, while the patient was fully under the influence of chloroform, and, unexpectedly, the head of the bone slipped into its proper place. Dr. Nathan Smith, Prof. of Surgery in Yale College, succeeded in reducing a luxated femur, by manipulating, with the thigh and leg used as a lever, after the usual plan had failed. It would seem from the report of this case, which, however, is made from memory by his son, Dr. Nathan R. Smith, that it was a deliberate and prearranged plan of procedure, though the considerations which led to its adoption are not mentioned in the record.

Chelius, in his work on Surgery, gives an outline of the views of four writers, who have proposed to effect the reduction of the dislocated hip by the hands alone of the surgeon and his assistants, and without the aid of pullies, or, indeed, of any forcible extension. Three of these gentlemen, Wattman, Kluge, and Rust, have described methods not very different from each other, which essentially consist of moderate extension with the hand, combined with movements of rotation of the axis of the limb, abduction, flexion, etc., but with the addition of force applied by a band passed around the upper part of the limb which is committed to an assistant, and, in the different forms of dislocation, is so applied as to lift the head of the bone from its unnatural resting-place, while the other movements made with the shaft of the limb give an opportunity to the muscles, stretched by the luxation, to draw the head back into the acetabulum. Another surgeon, referred to by Chelius, Colombat, speaks of a method which he has always employed with success, in which the patient stands at a table leaning forward with his chest upon it, and the surgeon, standing behind, bends the knees with one hand, and makes the various movements of the hip-joint, while, with the other hand on the ham, he makes gradual extension by pulling downward. By this manœuvre, he states that he dislodges the head of the bone, and without any force accomplishes its reduction.

In Casper's Wochenschrift for Nov. 1849, there is an account given by Dr. Fircher, of Cologne, of his mode of reducing luxations of the hip, which consists in flexing the femur on the trunk, and then making rotation of the limb, conjoined with odduction, or abduction, as the head of the bone is on one side or the other of the acetabulum. In the *N. Y. Journal of Medicine* for March, 1852, a Dr. Mayr is spoken of as having made use of this procedure of Dr. Fischer, without being aware of Dr. F.'s published statement.

Dr. Reid, in his paper, gives us an account of his observations on this subject, which appear to have been made entirely independent of any knowledge of the previous opinions of other writers. He speaks of his early impressions, on seeing the management of some cases of dislocated hip after the usual method, and gives his reasonings, and reflections, and experiments, on the muscular actions and the mechanical forces which were concerned, or might be employed, in the production and in the reduction of these displacements. He describes the steps by which he was led to the adoption of his mode of operation, and, what is most to the purpose, he gives five cases in which the operative procedure, to which he was led by reasoning, was successfully employed. It is no part of the object of this communication to criticise or to eulogize Dr. Reid's paper; but, having tested to some extent the value of the proposed plan, we feel that we owe to the profession a full statement of what appears to us to be a most valuable contribution to the art of healing. It is still less our intention to mete out the degree of praise which is due to Dr. Reid as the originator of the idea upon which the practice is founded. It would seem, from the little historical sketch given above, that the idea had occurred to others before it occurred to him, and that, therefore, the barren honor of being the first suggester of the idea, that luxations of the hip might be reduced by manipulations alone, does not belong to him. All who read his paper, however, will acknowledge, that to him belongs the higher honor, of having caused the idea to assume the shape and value of a fact, and out of the bare suggestion to have, by patient, ingenious, and long-continued investigation and experiment, deduced an available, and, as we think, an exceedingly valuable addition to the resources of Practical Surgery.

CASE I. The first opportunity which presented itself for the trial of the new method, was in the case of an Irish laborer, who was brought into the New York Hospital, November 30th, 1852, with a luxation of the right thigh. He had been struck, a short time before admission, by the cow-catcher of a passing railway train, and thrown some distance, and in his fall, probably, the accident was produced. The symptoms were those of the dislocation on the dorsum ilii, the head lying rather lower down and nearer the ischiatic notch than usual. The thigh was shortened about two inches, tended across the other, with the ball of the great toe of the injured limb touching the instep of the other foot, fixed in its position, and the head of the femur was felt in the position above described when the thigh was rotated on its axis. In addition to this injury, he had received a compound fracture of the left leg, three inches above the ankle, together with a good deal of bruising of other parts of his body. The patient was etherized to the extent of complete relaxation, and Jarvis's Adjuster was applied. It broke on the first trial of extension, and was laid aside. This mischance suggested the trial of Dr. Reid's plan, which was accordingly adopted. The operator, Dr. Buck, after bending the leg upon the thigh, gradually adducted the thigh, while at the same time it was being flexed upon the trunk. Carrying the limb thus bent at the knee, and strongly adducted, over the sound thigh, by a gradual sweep over the abdomen, and then slowly and steadily abducting the limb so as to carry the knee outward, making at the same time a rocking motion by moving the leg backward and forward, had the effect of dislodging the head of the femur from its new position, and making it approach the acetabulum; but it did not enter the socket. From the position above indicated, the limb was now brought down slowly toward a straight position, still kept in a state of forced adduction. This last manœuvre seemed to have a very powerful influence in forcing the head toward the acetabulum, but the whole proceeding was completed without success. It was observed, however, that the head had been moved a little higher on the dorsum than it was before. The same manipulation was now again practiced more deliberately and more carefully than before, and as the limb was being brought down abducted, we had the satisfaction of seeing and hearing the reduction effected, by the head of the bone slipping into its socket. All deformity had disappeared, and the motions were free in all directions. The other injuries were properly attended to, and the recovery from the effects of the luxation was rapid and satisfactory. He finally recovered from his compound fracture also, and left the hospital with a good leg and a perfect hip.

CASE II. An Irish laborer, aged 25, received an injury of his right hip, and a fracture of one of his clavicles, by being thrown from a railroad car, while it was in motion. He was received into the New York Hospital, December 8th, 1852, under the care of Dr. Halsted. On examination, the injury to the hip proved to be a luxation of the femur upward and backward on to the dorsum ilii. The patient was placed immediately under the influence of ether, and the reduction was attempted by a procedure nearly the reverse of that above described, in Case 1. The leg being flexed upon the thigh, the limb was flexed upon the trunk and carried up in a state of abduction, then across the abdomen, and being fully adducted, was in that state, brought down to the straight position. The effect of this mode of operating, which is almost precisely that said to have been employed by Prof. Nathan Smith, was to throw the head of the bone forward, under the anterior superior spine of the ilium, and it was quite evident that a very little more force in the same direction would have brought it upon the pubes. This plan was therefore abandoned, and Reid's manipulation was tried carefully and without the employment of much force. On the first trial it was successful, the bone being reduced with an audible snap, as the limb was being brought down in a state of abduction. The recovery was rapid and perfect, and he was discharged, cured, January 15, 1853.

Case III. Charles O. Merritt, a sailor, thirty-seven years old, of a stout, vigorous frame, was admitted to the hospital with a luxation of the femur, of twelve weeks' standing. Attempts had been made, by an excellent surgeon of this city, to reduce the bone, but without a satisfactory result. A very careful examination was given to the limb by all the surgeons of the hospital who were present, and all agreed that the head of the femur was thrown upon the dorsum of the ilium, in the usual situation; but some doubt existed whether there might not also be an injury of the acetabulum itself. The patient being fully etherized, Reid's manipulation was tried, and, on the first trial, failed, the head seeming to remain nearly in the position it occupied before the operation was commenced. Dr. Watson, under whose care the patient was, now made a second more careful effort, using more force in making all the movements, but being particularly careful to make forced abduction while bringing down the limb from extreme flexion to the straight position. As the limb was thus descending, slight rocking motions being at the same time employed, the reduction was suddenly accomplished, the head of the bone being felt, or heard by a great number of persons, to slip into its socket. The limbs being laid side by side, all deformity had ceased, and all present were satisfied that the reduction was complete and perfect. The patient's knees were bandaged together in the usual manner, and he was placed in bed with rather more care than usual, but in less than an hour it seemed as if the joint had lost its natural appearance again in a slight degree, and the apparatus was tightened. By next morning, however, it was too evident that the original displacement had again occurred, and to its fullest extent. This had taken place in spite of the greatest quietude on the part of the patient, who was a very intelligent, tractable person, and fully aware of the importance of keeping the joint unmoved. The manipulations were again tried several times, but without effect. The head of the bone seemed to move about freely in all directions, but could not be brought into the acetabulum. The limb was put up in the straight apparatus which we usually employ for fractures of the thigh, and extension, by the adhesive straps, was kept up so as to keep the parts, as nearly as possible, in proper position. A good deal of stiffness and swelling of the joint followed, which, however, subsided, and he was allowed to go about on the 30th January. He finally gained about as much use of the joint as if there had been a fracture of the cervix femoris. I am informed by Dr. Buck that he has since gained a very excellent use of the limb.

Case IV. John Kelly, a laboring man, aged twenty one years, was admitted May 22d, 1853, having been knocked down by a horse-car, by which a luxation of the left hip had occurred into the ischiatic foramen. The limb was shortened about one inch, toes turned inward, and the head of the bone felt in its new situation. The reduction was attempted by the mode described above, the man being fully relaxed by ether. The effect of the first attempt was to throw the head on to the obturator foramen, making the limb longer than the other, and producing the deformity characteristic of that dislocation. From this point, by a slight alteration of the movement, the head could be made to slip back to its original position. Between these two points it could be made to play backward and forward, but would not enter its socket. Dr. Post, in whose charge the patient was, then employed the usual mode of reduction, from the foramen ovale, viz., the extension of the limb, combined with a lifting of the head of the bone over the edge of the acetabulum, by the help of a folded sheet passed round the upper part of the thigh. This proved successful, without resorting to the pullies. In this case the cure was very slow, and he left the hospital with some degree of pain and swelling about the joint. I learned that an abscess formed in or about the joint, which was opened, and when I saw him a year after, there was every appearance of seated morbus coxarius.

Case V. Michael Delaney, a boy aged eight years, was admitted into the house, June 29th, 1853, having received very severe injuries in falling from a ladder, at the height of the third story of a house, to the ground. There was found to be a bad compound fracture of the right thigh, and simple fracture of the left. When laid upon a bed, and his clothes removed, the right thigh, which was the seat of compound fracture, was found to be in an extraordinary position. It lay obliquely across the abdomen of the boy, with the leg and foot lying up by the axilla of the left side. On examination, it was discovered that this singular position was rendered possible by the fact that the head of the femur was dislocated backward and upward, on the dorsum ilii. The house surgeon, to whose care the case fell on admission, took the injured limb in his hands and very carefully carried it over the abdomen to the right side, and then abducted it and brought it down toward the straight position, thereby completing the steps of Reid's manipulation, which accident had already commenced. In doing this the head of the bone slipped into its place, and the hip gave no further trouble. The fractures of both thighs went on favorably toward a cure, and he was discharged well, August 23, 1853.

Case VI. John Gallagher, twenty-eight years old, a stout Irish laborer, was brought into the hospital about half an hour after he had fallen from his cart while intoxicated. He was found to have a dislocation of the left femur upon the dorsum ilii, and was immediately sent to the operating theatre and placed fully under the influence of ether. I then proceeded to employ the manipulation above described, but as we were completing it by bringing down the limb strongly abducted, it became evident that the head of the femur was about to slip below the acetabulum into the foramen ovale, as had occurred in Dr. Post's case, No. 4. The movement was, therefore, reversed, and again more carefully tried, with the same result. On the third trial, with the view of avoiding this tendency of the head to be forced below the acetabulum, I brought down the limb in a state of very slight instead of very strong abduction, making the movement very deliberately, and accompanying it with the usual rocking motions of the limb on its axis. This plan was perfectly successful, and the bone slipped easily into its place, and the cure was rapid and perfect. All the movements in this case were made with the greatest facility, and without the necessity of employing any more force than the weight of the limb required.

After the reduction in this case, I made a dissection of the hip-joint in a recent subject. I had previously made several dissections with reference to the action of the different muscles and their condition after luxation. In this instance, however, I removed all the muscles, leaving the capsular ligament only, and then endeavored to dislocate the head of the bone. I first tried adduction, and carried the limb so forcibly over the abdomen that the knee touched the anterior surface of the thorax, but without producing luxation. On making more violent efforts in the same direction, the cervix fractured or rather cracked across within the capsule, and soon after the ligament itself tore across at its superior and posterior part, just opposite the point of yielding of the cervix. The laceration was directly across the ligament, and occupied about one-half of its circumference. As soon as this took place, the dislocation was easily effected. The neck of the femur and the trochanteric portion of it were now seen to be kept in their place by the untorn portion of the capsular ligament, which acted as a sort of fulcrum, upon which, by using the limb as the long arm, we could make the head, as the short arm, move about in any direction upon the surface of the dorsum of the ilium. On making the manipulations for its reduction, the head was observed to pass behind the acetabulum until it reached its lower margin, and if the thigh was brought down slightly abducted, it slipped easily over this least elevated part of the margin into its socket. If, however, it was brought down very strongly abducted, the head, instead of passing into, went below the acetabulum, and passed on to the foramen ovale, as it was about to do in Gallagher's case, No. 6, and as it did repeatedly do in Kelly's case, No. 4. Still further, we observed that after we had reduced the bone it slipped very easily out of place again, and this was explained by finding that in the reduction, the head had not gone back through the rent in the capsular ligament, but, remaining outside, had pressed the capsule before it into the acetabulum. This, of course, prevented the complete reception of the head of the bone into the socket, and permitted it to slip out again with the greatest facility. This entanglement of the head in a button-hole opening of the capsular ligament is noticed by many surgeons as an obstacle to complete reduction by the old plan with the pullies, and I do not perceive that the new method offers any advantages in this particular. Might not this have been the condition of things in Merritt's hip, case No. 3?

Case VII. Francis Cotbunger, an Irish turner, was admitted to the N. Y. Hospital, December 12th, 1853, with a dislocation of the right hip. The accident had occurred about four weeks previously, and had been treated in the country, as a sprain, by leeches, etc. The limb was lengthened, the toes everted, and the whole limb stood off from the body abducted, and slightly flexed, symptoms which clearly showed that the head of the bone was upon the foramen ovale. The patient being fully brought under the influence of ether, a manipulation the reverse of Reid's, was employed by Dr. Halsted. The leg being bent upon the thigh, the thigh was gradually flexed upon the trunk until the knee touched the thorax. The limb was then brought down, forcibly adducted, into the straight position. By this the head of the femur was moved from the foramen ovale on to the dorsum ilii. Being in this situation, Reid's method was adopted, with the effect, however, of bringing back the head to its original position on the foramen ovale. By a repetition of the first manœuvre, it was again thrown on the dorsum, and from there, by Reid's plan, again thrown back upon the foramen. After repeated attempts, the bone was finally reduced by the pullies from the dorsum in the usual way, this being the only instance in which the pullies or Jarvis's Adjuster have been used, since our attention has been called to the new plan. It will be noticed, however, that the limb was every time brought down in a state of forced abduction; the moderate abduction found successful in Gallagher's case, No. 6, was not tried. A good deal of swelling and pain in the joint followed these various operations. He was up and about, however, by the 5th January, and on the 13th he was discharged, cured.

Case VIII. Hugh Doyle, a sailor, 22 years old, was admitted to the hospital, April 22d, 1854, with a dislocation of the left thigh upon the foramen ovale. The limb was in a position of abduction, rotated outward, with the foot resting on its outer edge. The muscles arising from the pubes and ramus of the ischium were very tense, adduction was impossible, and the prominence of the trochanter was gone. From this deformity, the position of the head, with regard to the acetabulum, was clear. He was also suffering from other severe injuries, which had been produced by the fall, in which the hip was luxated. He was put fully under the influence of ether, and the reduction was first attempted in the usual way, by Dr. Buck, by direct extension of the limb in the direction of its axis, while at the same time the upper part of the thigh was lifted forcibly outward by means of a sheet folded round it. This had not the desired effect, and would have required the pullies to make it effectual. Another manipulation was then tried. The leg was flexed upon the thigh, and the thigh upon the trunk. The foot was then pressed forcibly outward, thus rotating the thigh inward on its axis, and giving the head of the bone an inclination outward toward the ischiatic notch. The pelvis at the same time was held firm by assistants. This was repeated twice, each time with the effect of causing the head to move considerably from its resting-place, but without entering the socket or going over to the notch. On the third time of making the manipulation, the thigh was brought down from entire flexion to a little below a right angle, and while in this position the foot was again forced outward as before. This plan proved successful, and the head of the bone slipped into its proper place in the acetabulum. This was accomplished without any considerable force or difficulty. The cure went on favorably, and without unusual symptoms.

Case IX. Fritz Frieze, aged 38, a German sugar refiner, came into the Hospital, May 23, 1854, with a luxation of the left thigh, which had occurred fourteen days before from a fall. The surgeon who first saw him pronounced it a mere contusion, and had allowed him to walk about, which he was able to do without much pain, though, of course, with a very great halt in his gait, and with great labor in the movements of the injured hip. As he stood erect, the left thigh inclined outward and forward from its proper direction, the toe of the injured limb touching the floor about ten inches from the other foot. The limb was an inch longer than its fellow, and the adductor muscles were tense. Adduction was impossible. The trochanter was fallen in toward the pelvis, and thrown backward and downward. On making rotation, the head of the bone could be indistinctly felt moving on the foramen ovale. After having the patient fully relaxed by ether, I made some free movements of the limb in all directions, with a view of breaking down any adhesions which might exist. I then took hold of the ankle of the injured limb with the right hand, while I supported the thigh with the left; I flexed the knee to a right angle, and moving the thigh a little forward, to relax the tense adductor, I forced the foot inward, making, at the same time, a rocking motion of the whole limb on the pelvis. My idea was, by rotating the thigh outward, to throw up the head of the bone toward the acetabulum, from which it could not be distant more than an inch. This manœuvre was not successful, though carefully and repeatedly tried. We then tried the plan adopted by Dr. Buck, in Hugh Doyle's case, No. 8, with the effect of throwing the head upon the ischiatic notch. By reversing the movement, the head was easily thrown back to its previous position on the foramen, and the first manipulation was again tried, combined with a movement of strong adduction in a direction which carried the knee behind the other one. This, accompanied with the rocking motion, which we have found useful in all these replacements, accomplished the reduction on the first trial, and without the slightest violence or excess of force. The head slipped in with an audible snap, and the limb assumed its natural shape and position. The cure was rapid and perfect.

The two last cases were experimental, as we had no knowledge of any recorded precedent which gave us any assistance in their management. We were led to adopt the modes of manipulation, simply from reasoning on the effect which the movements of the thigh would produce on the position of the head of the femur. An attentive examination of the skeleton, with the head of the femur placed on the foramen ovale in the position it assumes when luxated, will show that while the head lies upon the foramen, the neck lies along the side of the pelvis in such a manner as to bring the trochanter about opposite to, and a little below, the acetabulum. If, now, we hold the trochanter fixed with one hand, and make the two movements described in Cases 8 and 9, it will be observed that the head can be brought into place by both of them; but, at the same time, it will be observed that, when the trial is made by rotating the thigh inward, thereby causing the head to move outward, the head, in order to reach the socket, has to pass through a sweep of ¾ of a circle, and then crosses the lip of the acetabulum at a point where it is very prominent and very difficult to surmount. If, on the contrary, the rotation of the thigh is made outward, so as to throw the head inward, it has only a space of about an inch to pass through in order to slip into place, and the portion of the brim over which it passes is much less elevated and prominent. The movement of adduction in a direction a little backward, as employed in Case 9, has evidently the effect to lift the head over the edge of the acetabulum when the rotation has brought it to that point. It will also be noticed that in the outward sweep of the head, it has, in order to get up to the acetabulum, to pass by the ischiatic notch, into which it is exceedingly prone to slip, as actually occurred in Case 9, and also in Case 4. For these reasons, I am disposed to consider the manipulation employed in Case 9 to be the correct and most effectual one in this form of luxation, and that though in a recent and favorable case the outward movement of the head might succeed, yet that in a difficult or long-standing case it would fail entirely, or be attended, if successful, with a great degree of violence and laceration. This is a point, however, which can only be settled by more ample experience and careful experiment. From the comparative rarity of this form of luxation, this experience can only be slowly obtained, and we should feel greatly indebted to any gentleman who may have an opportunity of managing such a case by manipulation, if he would transmit to us the results of his experience.

Case X. Dr. Dewitt C. Peters, late house-surgeon of the N. Y. Hospital, now of the U. S. Army, writes from Pawnee, Fork River, Kansas Territory, under the date of Sept. 24, 1854, "I have had one case of dislocation of the head of the femur into the ischiatic notch, which I reduced immediately after it occurred, by putting the man under the influence of ether and employing the method of Dr. Reid." The man had been thrown violently by an unruly mule he was attempting to harness.

The following letter from Dr. W. Parker, Professor of Surgery in the University of the State of New York, adds two more valuable cases to our list.

New York, Nov. 10, 1854.

Dear Sir:—In compliance with your request, I send you an account of two cases of luxation at the hip, which I reduced after the plan proposed by Dr. Reid, of Rochester, N. Y.

Case XI. July 11, 1853. I was called in the evening, to Mr. P—— C——, a contractor, hale, muscular, and forty years old. I found him lying on the carpet, complaining of pain. He was thrown from his carriage, by the running away of his horse. On examination, I found the left limb an inch and a half shorter than the other, and the foot turned inward. The head of the femur was in the sacroischiatic notch.

I put him *fully* under the influence of chloroform, and proceeded to reduce the thigh. In my first attempt I carried the head of the bone into the foramen thyroideum. By a slight movement and without force, the head of the bone passed up to the notch. I at once made the second trial, and carried the bone into place with the utmost ease. The patient was soon at his business.

Case XII. November 1, 1854. Mrs. K., aged forty-four, fell some eighteen feet, injured the brain and luxated the left thigh. I saw her at two P. M. Advised to dress the head, get up reaction, and I would be back in a few hours to reduce the limb. This was a luxation upon the dorsum. She was strong and muscular.

I found her in the evening warm and sensible. I applied chloroform and proceeded to reduce. I accomplished it after the plan of Dr. Reid, without any violent effort and in a most satisfactory manner. I am prepared to say, the profession are under great obligations to Dr. Reid for the plan of treatment which he has suggested and demonstrated in the reduction of luxations at the hip-joint. With the aid of *an* anæsthetic the plan is perfect. W. P.

Case XIII. Sam. Gordon, aged thirty-seven, Ireland, laborer, was brought to the N. Y. Hospital, November 22, 1854, with a luxation of the right hip.

and a fracture of the right leg. The accident had occurred immediately before admission, from the falling of the wall of the gas-house. The head of the bone was found on the dorsum ilii, rather low down toward the ischiatic notch.

Nov. 24. The leg having been dressed firmly with pasteboard splints, so that it might not receive injury during the reduction of the hip, the patient was fully etherized and Reid's plan of reduction was tried by Dr. Watson, twice or thrice without effect. This plan was abandoned, and the pullies were faithfully and for a long time employed. The head descended, but would not pass into its socket. Thinking that Jarvis's Adjuster would give more control over the movements necessary in replacing the bone, it was applied, and with it the head of the femur was brought down so that it seemed ready to slip in, but on cutting the cord it was found unreduced. It was thoroughly tried until the steel shaft bent, but without success. Under these discouraging circumstances, the pullies were again about to be applied, when it was thought best to give Reid's plan one more trial. The manipulation was performed twice, without success. The third time it was done more deliberately and more carefully, and when the head of the bone was brought to the lower edge of the acetabulum, it was retained there for a time and the rocking motion performed in all directions, and when all seemed loose and free, the abduction was increased without any jerk or sudden motion, and the limb brought down thus strongly abducted toward the straight position; as this was done, the head of the bone slipped into its place with an audible snap.

From the small number of cases here presented, even with the addition of those recorded in Dr. Reid's paper, I do not consider that we can obtain data sufficient for the final appreciation of the new plan of reducing luxations of the hip-joint. Still they seem to be sufficient to show us that the plan may be safely, easily and successfully applied to a certain number, and probably to a very great proportion of all the cases which occur. The cases which are related embrace every instance of dislocation of the femur which has presented itself in the Hospital during the last two years, and a reduction was always effected without the aid of the pullies, except in the one case of Dr. Halsted, No. 7, and here a modification of the manipulation, which had previously proved successful, was not tried. In Dr. Post's case, No. 4, the reduction was finally effected from the foramen ovale by the usual mode, though without the pullies, but it was brought, by the manipulation employed, to this point from the ischiatic notch, from which it would surely have required the pullies to displace it on the old plan. It is to be observed, too, that our experience has been progressive, and while, at the time Case No. 4 occurred, we did not know how to reduce the bone from the foramen ovale by manipulation alone, that we have since learned the true method of doing it, if our experience, in Cases 8 and 9, can be relied on. Though we have failed, then, in reducing, by the new plan, two cases out of the thirteen, yet I think that the record shows good reason for believing that all might have been so reduced, if we had known at the time as much as subsequent cases have taught us. The applicability of the plan to cases of long standing, is well shown in Merritt's case, No. 3, where twelve weeks had elapsed from the time of the accident, and in which, though not permanent, the reduction was effected with comparative facility.

With regard to the rationale of the process, most of those who have written on this matter are in the main points agreed. The head of the dislocated femur is retained in its new position by a mechanism which does not exist in any other joint, and which is produced by the fact of the muscles not being inserted into the head, but into the trochanter, nearly three inches from the head, and that from this point of principal muscular insertion the neck goes off at a large angle from the axis of the shaft. From this, it happens that when the head of the femur is thrown out of its socket, the trochanter no longer stands out more prominent than before, but being held firmly by the muscles which are inserted into its base, it is prevented from rising any more than enough to let the head out of the acetabulum, while the head and neck, slipping to the one side or the other, are found lying in such a manner that the side of the head, neck and trochanter are in contact with some part of the outer surface of the pelvis, varying, of course, in the different forms of luxation. This being borne in mind, it will be clear that any attempt at reduction, which merely brings the head of the bone to the acetabulum, will not succeed in making it enter that cavity, because of the lying-down position of the neck and trochanter against the side of the pelvis. We need, therefore, not only to bring the head over the socket, but at the same time to raise up the trochanter and neck so as to allow the head to enter. Now, in the ordinary methods of reduction, this raising up of the trochanter, so as to put the neck in the proper direction for the head to enter its socket, is done first, by the action of the pullies, and the approximation of the head to the socket is done second, by the continuation of the extension. This raising of the trochanter is, of course, opposed strongly by the muscles inserted into its base, causing the head to be pressed more and more firmly against the pelvis, and increasing the friction, and thereby causing, by far, the greater part of the difficulty in bringing down the head to the level of the acetabulum. It is in this, principally, and I am myself disposed to say only, that any active muscular contraction opposes the reduction of a dislocation of the hip-joint. True, the large muscles around the joint are thrown into action as soon as extension is made; but this is an action excited by the extension and, that it is a very feeble opposing force, is evidenced by the facility with which these muscles give way to the force of a single unaided arm, when a fracture of the neck of the femur is concerned, in which, of course, none of the friction alluded to can occur. This comparative action of the muscles, in fracture and in dislocation, is very strongly and appropriately insisted upon by Dr. Reid.

The process by manipulation avoids this main difficulty, and as it were, eludes the opposition of the muscles. The trochanter, being fixed by the insertion into its base of the pyriformis, the two obturators, the gemelli, and the upper part of the quadratus, acts as a fixed point or fulcrum, upon which, by moving the limb, the head of the bone can be made to describe a circle round the fulcrum. When we remember that this fulcrum is not, strictly speaking, a fixed point, but has a certain degree of motion of its own, we can easily see how, by means of this movable fulcrum, the head of the bone can be placed, by varying the motions of the limb, on almost any point within two inches around the acetabulum, and, of course, over the acetabulum itself. If this manipulation is made in such a way as not to raise the trochanter from lying against the pelvis, then, when the head comes over the acetabulum, a slight rotation, such as is given by the rocking motion employed, will sufficiently raise the trochanter to let the head slip in without provoking to opposition the trochanteric muscles, and if the movements be made in such a direction as to relax the stretched muscles, the whole may be accomplished without calling forth the slightest muscular opposition from the beginning to the end of the procedure. This principle, in its application to the different forms of dislocation, presents some variations. In the dislocations on the dorsum, and on the ischiatic notch, for their mechanism is for our purpose identical, the principle has its best illustration; and if any one will take the skeleton or the dead subject, and go through the process, he will perceive that, by adduction, the tense rotators are relaxed, and that, by flexion of the thigh upon the trunk, the head is caused to pass down behind and below the acetabulum, and then, by carrying the knee out so as to abduct the limb, that the head comes toward the lower portion of the acetabulum where its margin is least prominent. At this point, I wish it to be observed, that our mode of procedure varies a little from Dr. Reid's. He recommends, when the head is brought by abduction close to the lower edge of the acetabulum, that, by the rocking movement already described, it be caused to slip in. This is well, and will probably answer in many cases, but it failed us so completely from the first, that we were led to add the bringing down of the thigh to the straight position in a state of abduction, still keeping up the rocking motion, and it has been uniformly in the act of thus bringing down the limb, that the reduction has been accomplished. On looking at the parts in the dead subject, it will be seen that this movement of the limb, when the head has reached the lower margin of the acetabulum, tends directly to roll the head upward over the edge and into the socket. The mechanism of the reduction from the foramen ovale has already been alluded to. I do not know of any case of reduction from the pubes.

If the proposed plan should prove, on further trial, to be as successful and as free from danger as it thus far has been, one most valuable feature of it, as a surgical resource, will be its availability. Wherever a surgeon, with his bottle of ether, can go, there the dislocated hip can be reduced, without instruments, without appliances, without assistants. I well remember finding myself, in the year 1848, in a country village in Vermont, in consultation on a case of dislocated hip of some weeks' standing, in which it was difficult for us to persuade the patient's friends that there was any thing more than a sprain of the joint. It was late on a cold autumn day, and we were about twenty miles from any place where pullies could be obtained; but, nevertheless, we made such extempore arrangements as we best could, and by the help of an old tackle-block and some bed-cord, with counter-extending bands of hanks of homespun yarn we proceeded to attempt the reduction. If we had been heartily seconded by the friends and family, we could, I have no doubt, finally have succeeded, even with our clumsy apparatus; but the doubts and hesitations on their minds, and their unwillingness to allow the patient, a stout, muscular young woman, to be so long and so repeatedly subjected to our unsatisfactory attempts, finally decided the case against us, and we were dismissed without thanks or fee, and the poor girl's hip has never been reduced to this day. I have often thought since, how different an aspect matters would have worn, if we could have brought the manipulation to bear upon the case, and how different a result might have been obtained for the unfortunate patient. No better illustration, however, could be given of the availability of this operation than that given in the letter of Dr.

Peters, giving an account of Case 10, in which he says, that the accident occurred in the heart of Kansas Territory, five hundred miles from the nearest fort. If the poor man had depended upon pullies for the reduction of his dislocation, how much delay and suffering must he have undergone, from which he was saved by the simple fact that Doctor Peters was familiar, during his hospital course, with the management of these cases by manipulation, and was therefore able, on the spot, to effect with ease a reduction which every day's delay would have rendered more difficult.

Every thing in our experience thus far seems to indicate that this method of reducing luxations is as safe, if not safer, so far as the integrity of the joint and its after usefulness is concerned, than the reduction by forcible extension with the pullies. The method is not, however, without its dangers, and these mainly arise from the immense amount of force which can be exercised by acting on the long arm of so powerful a lever as the whole limb, while the short arm has at most a length of three inches. By the inconsiderate or misdirected action of this lever force, one of three accidents might be produced: either the muscles holding the trochanter, and thereby forming the fulcrum on which the power is applied, might be torn from their attachments; or, if they held fast, the tissues, round and among which the head passes, in its movements, may be extensively lacerated or contused; or, lastly, it seems to me very possible that the neck of the bone might be broken by too violent abduction, forcing it against the side of the pelvis. Though happily these dangers are thus far only theoretical, yet the anatomy of the part shows that they are real, and each one of them might have most serious consequences. It would not be possible to lay down any specific rules whereby these dangers are to be avoided, except by insisting on that almost universal law of surgical manipulation, that every thing is to be done with gentle moderate force, and never with sudden violence. As far as my recollection serves me, and I myself assisted in almost every case reported, we never have accomplished any thing by proceeding in a direction where great force was required to continue the movement, but have always succeeded by finding a direction in which the mere continuance of the movement, without force, has brought the head into the proper position. It will be noticed, by looking over the cases, that in many of them, before the head went into its socket, it slipped about on the outer surface of the pelvis, taking sometimes one and sometimes another of the four positions usually spoken of as the four forms of luxation of the hip. This in some instances happened several times, and in the two instances of failure, this change of position was all that could be accomplished. Now, it cannot be doubted that this extensive movement of the head of the bone must be attended with a corresponding amount of laceration and displacement of the tissues among which it passes, and although the mischief thus done may be confined to the areolar tissue in the muscular interspaces, still it is an injury which may add to the dangers of after-inflammation, and is, if possible, to be avoided. I suppose this can only be done by defining more accurately the precise method of procedure to be adopted in each case, so that no experimental or ineffectual trials shall have to be made, but the operator shall be able at once to do exactly what is necessary to bring the head of the bone to the point desired.

The above account had been prepared for the press, with the exception of Case 13, which has since occurred, when a case presented itself which offers some points so important in their bearing upon what has already been said, that I add the history here by itself, rather than incorporate it with the other cases.

Case XIV. Patrick Barry, aged 42, was admitted to the New York Hospital, Oct. 23, 1854, with a dislocation of the left femur, which had occurred seven weeks previously, by a fall from a rail-car while it was in motion. The symptoms were unequivocal, the limb being shortened $1\frac{3}{4}$ inches, the ball of the great toe resting on the instep of the sound foot, and the head of the bone being distinctly felt upon the dorsum of the ilium. The patient was a man of good muscular development, but the injured limb was somewhat wasted and flabby from inaction. Two days after admission he was put under the influence of ether, and Reid's manipulation was tried. The head descended as usual, until it came opposite to the lower margin of the acetabulum, but from that point, as the limb was brought down, it slipped on to the foramen ovale. The manipulation was repeated several times, with all care, varying the degree of abduction on the various trials, but without success. It was impossible to make the head rise over the lower border of the acetabulum so as to slip into its place. After numerous thorough and careful trials, the manipulation was abandoned and the pullies ordered to be applied. Before this was done, it was thought best to place the head of the bone on the foramen ovale, and from that point to try and reduce it by the usual method recommended by Sir Astley Cooper. The head was accordingly placed on the foramen, and while the upper part of the thigh was grasped by an assistant and lifted strongly outward, I took hold of the ankle and made extension and adduction. The head seemed not to move at all under this force, and while making strong adduction a crack was heard, every thing became loose about the joint, and on examination it was evident that a fracture of the cervix had taken place, leaving the head in the foramen ovale. There was nothing further to be done but to put the limb up in the straight apparatus, hoping that, if we could obtain union, he would have as useful a limb as those ordinarily left by fracture of the cervix, and certainly a better limb than if the dislocation had been untouched. Thus far, Nov. 25, every thing has gone well, and promises union, with a shortening of about an inch. I am sorry that we must accept this case as one of failure of the new plan after what we considered a fair trial; for myself, however, I do most profoundly believe that it failed simply because we have not yet learned enough about the manipulation to adapt it to the condition of parts concerned in this particular instance. That we shall yet acquire that knowledge, I see no reasonable ground to doubt. With regard to the fracture of the cervix, we were all surprised at the slight amount of the force which was cempetent to produce such a mortifying accident. It adds double force to the caution given above, when speaking of the possibility of that accident, and it is not a little remarkable that the paragraph containing that caution was written on the very morning of the day when the production of the fracture verified the necessity of the warning. Dr. Watson, in a note to me, speaks of a fact which he says, "I have on undoubted authority, viz.: from one of the professors in the School of Medicine in Toronto, Ca., that an accident, similar to that of Case 14, occurred in that city, while the surgeon was attempting to reduce a luxation of the hip by Reid's method." Finally, it must be observed that the new plan is entitled to none of the blame of the fractured cervix. The accident took place after Reid's manipulation was abandoned, and while we were attempting the reduction according to the old established and classical method.

NEW YORK, Nov. 30th, 1854.

